# Modeling and Performance Analysis of a Liquid Desiccant Cooling and Dehumidification System Using ITSO-TCN-BiGRU-SA

**DOI:** 10.3390/s26144539

**Published:** 2026-07-17

**Authors:** Xianhua Ou, Xinkai Wang, Zheyu Wang

**Affiliations:** College of Information Engineering, Zhejiang University of Technology, Hangzhou 310023, China; 18868447091@163.com (X.W.);

**Keywords:** liquid desiccant, TCN, BiGRU, cooling and dehumidification, performance investigation

## Abstract

Liquid desiccant dehumidification, an energy-efficient technology for air humidity control, has gained significant attention in recent years. In this study, the air temperature and humidity prediction models of liquid desiccant cooling and dehumidification (LDCD) system are built based on the proposed ITSO-TCN-BiGRU-SA. In the proposed model, the TCN is employed to obtain local features within sequence and improve the learning ability of temporal dependencies; BiGRU strengthens the model through global and bidirectional contextual relationships; self-attention mechanism assigns different weights to each time step. The ITSO algorithm, which combines the nonlinear adaptive weights and Levy flight strategy, is proposed to find the optimal hyperparameters of network. Accordingly, the model prediction accuracy is improved. Through comprehensive comparative analysis with other models under a series of experiment results, the superior performance of the developed models was systematically validated. Furthermore, based on the model predictions and experimental results, a comprehensive analysis was performed to systematically investigate the impact of system inlet parameters on cooling and dehumidification capacity and efficiency, which can provide valuable guidance for system control and operation.

## 1. Introduction

Air cooling and dehumidification are essential for maintaining indoor thermal comfort and ensuring high air quality. However, these processes performed by air conditioning systems account for a significant portion of building energy consumption. In Singapore, building air conditioning systems consume approximately 23% of the nation’s total energy [1]. In the field of air conditioning research, energy efficiency and system capacity are two key areas of focus. Since the development of the first liquid desiccant dehumidification system (LDDS) in 1955 [2], its high energy efficiency and exceptional dehumidification capabilities have spurred extensive research. Over recent decades, the LDDS system has been studied in-depth in system design [3,4,5], system modeling [6,7,8], performance analysis [9,10,11] and optimization [12,13,14].

An accurate model serves as the foundation for performance analysis and optimization of the LDDS system. To this end, various models have been established, which can be classified into three main categories, i.e., the grey-box model, physical-based model and data-driven model. Among these, physical-based models are derived from fundamental thermodynamic principles and physical theories, integrating detailed information such as the structural configurations of system components and thermophysical properties of working fluids. Liu et al. [15] developed a state-space model for regenerator, incorporating thermodynamic properties of both the desiccant solution and packing materials. Chen et al. [16] introduced a finite-difference model for packed-type liquid desiccant equipment, effectively capturing the coupled heat and mass transfer processes, with model predictions demonstrating strong agreement with experimental data. In [17], a dehumidification performance model for an internally cooled dehumidifier was established, incorporating an analysis of the influence of the thickness and shrinkage of liquid falling film. Besides this, a physics-based modeling approach has been adopted by studies [18,19]. The grey-box model is derived from physical or thermodynamic principles, while the model parameter is determined through the parameter identification method with experimental measurements. Shen et al. [20] introduced a novel desiccant regeneration system and established a heat and mass transfer model to describe the liquid desiccant regeneration process. The numerical predictions demonstrated good agreement with experiments, maintaining a maximum relative error margin of 10.03%. Additionally, the system regeneration performance under different working conditions was analyzed and compared. Wang et al. [21] proposed a hybrid model for a batch-wise LDAC system. Meanwhile, the system’s dehumidification performance was also investigated. In [22], a dynamic model for a regeneration system operating under vacuum conditions was established on the basis of mass and enthalpy balance principles. The model predictions aligned well with experimental measurements. Furthermore, the transient regeneration performance of the system was investigated under step changes in the inlet temperatures of chilled and hot water. Similarly, Wu et al. [23,24] and Li et al. [25] adopted comparable modeling approaches in their respective studies. Physical models are built upon the principles of thermodynamics and mass transfer, offering good interpretability. However, their construction often relies on internal system parameters that are difficult to accurately obtain in complex boundary conditions, and they incur high computational costs, making them less suitable for real-time applications. Gray-box models simplify the structure by identifying some parameters through experimental data, but their predefined model frameworks may face limitations in generalization when dealing with the highly nonlinear and strongly coupled dynamic characteristics of the system. In contrast, data-driven models, especially those based on deep learning, demonstrate unique practical advantages: they do not require predefined complex mechanistic equations and can autonomously learn the intricate nonlinear mapping and dynamic dependencies between multidimensional input variables and target outputs directly from historical time-series operational data. This approach avoids the cumbersome process of mechanistic derivation and parameter acquisition, resulting in higher development efficiency. Moreover, a well-trained data-driven model is capable of performing rapid and accurate forward predictions, which provides a feasible technical pathway for its integration into real-time optimization frameworks.

As an effective modeling approach, data-driven modeling has been extensively studied and applied in various system modeling applications. These models are constructed using experimental datasets obtained under a range of working conditions, enabling accurate prediction of system output variables from corresponding input variables. In recent years, neural network-based modeling methods have attracted considerable attention in the field of building air conditioning systems. Gandhidasan and Mohandes [26] proposed an artificial neural network (ANN) model of a dehumidifier, and the system’s performance was investigated. Mohammad et al. [27] built a dehumidifier model by using the ANN approach; the dehumidification effectiveness and water condensation rate were then predicted. The relative errors between model predictions and experimental results were within 10%. In [28], a long short-term memory (LSTM) network was employed to predict indoor air temperature in a multi-loop central air conditioning system. The results revealed that the LSTM model outperformed the recurrent neural network (RNN) model in terms of predictive performance. To further enhance prediction accuracy, hybrid models integrating multiple deep learning modules have gained increasing attention. Elmaz et al. [29] developed a CNN-LSTM hybrid model for indoor air temperature prediction. In this architecture, the convolutional neural network (CNN) serves as a local feature extractor, with extracted features subsequently fed into the LSTM pipeline for multi-step temperature forecasting. Verification results indicated that the proposed model maintained stable performance across prediction periods of up to 2 h ($R^{2}$ > 0.9). The above model also has some limitations. The hybrid model contains multiple adjustable hyperparameters, which will have a direct impact on the prediction results. How to find the most suitable hyperparameters for the model is a key issue. Therefore, integrating optimization algorithms into hybrid models is also the current mainstream trend. Wu et al. [30] combined the LSTM with the Genetic Algorithm (GA) to optimize the ventilation control system of residential buildings. They used the GA to automatically search for the optimal hidden layer structure and spatial dropout rate of the LSTM. This optimization method significantly improved the model’s prediction accuracy. Guo et al. [31] considered the feasibility of optimizing the MLP model with four metaheuristic algorithms, including salp swarm optimization, whale optimization, spotted hyena optimization and wind-driven optimization. The results demonstrated significant improvements in prediction accuracy, with the heating load prediction accuracy increasing from 83.85% to 92.48% and the cooling load prediction accuracy rising from 60.61% to 89.29%. Furthermore, a data-driven modeling approach was also adopted in studies [32,33,34].

**Figure 1:**
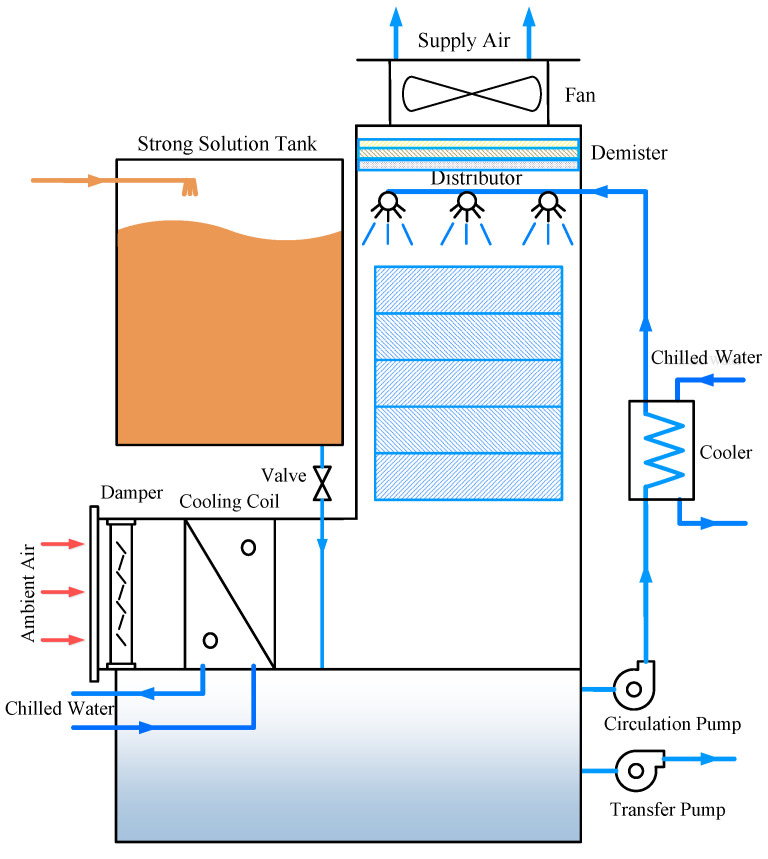
Schematic diagram of the LDCD system.

In LDDS, the regeneration of the desiccant is a major contributor to overall energy consumption [35]. To address this issue, a two-stage liquid desiccant cooling and dehumidification (LDCD) system, as illustrated in Figure 1, is constructed. The system aims to reduce the dilution rate of the desiccant solution in the dehumidifier, thereby decreasing the frequency and energy consumption associated with solution regeneration. Although the LDCD system demonstrates higher energy efficiency compared to conventional LDDS, its energy consumption can be further minimized through optimized operational strategies. To enable precise energy optimization of the LDCD system, an accurate system model must first be developed. The LDCD system involves complex two-stage heat and mass transfer processes, which require a robust modeling approach. In this study, we propose a novel hybrid prediction model integrating the temporal convolutional network (TCN), bidirectional gated recurrent unit (BiGRU), self-attention mechanism (SA), and improved tuna swarm optimization (ITSO) to accurately predict the supply air temperature and humidity. The TCN layer captures temporal dependencies and local patterns within the input sequence via convolutional operations. The BiGRU layer, connected to the TCN layer, operates at a higher level to model bidirectional relationships and long-range dependencies. The SA mechanism dynamically identifies critical features in the sequence and assigns adaptive weights to each time step. Meanwhile, the ITSO algorithm optimizes the model by identifying the most suitable hyperparameters. Finally, the proposed ITSO-TCN-BiGRU-SA model is rigorously evaluated and compared with other models to demonstrate its effectiveness. Additionally, based on the model predictions, the influence of system inlet parameters on cooling and dehumidification performance is analyzed. The findings of this study provide valuable insights for optimizing the performance of the LDCD system and guiding its operational control.

## 2. Methodology

As illustrated in Figure 1, the LDCD system comprises two primary components, namely a dehumidifier and a cooling coil, where the mass and heat transfer process occurs. In this system, outdoor air is drawn by fan into the cooling coil and dehumidifier. At the same time, chilled water is circulated by a water pump into the cooling coil and cooler, while the desiccant solution is pumped by a solution pump through the cooler and dehumidifier. In the cooling coil, air flows through the gaps between fins and undergoes heat exchange with the chilled water. As a result, the air is cooled and the humidity gradually reaches saturation. As the air temperature continues to drop, the moisture in the air is condensed and precipitated, achieving the first stage of cooling and dehumidification. The chilled water is heated and then sent back to the chiller system. The desiccant solution also undergoes a heat exchange reaction with chilled water in the cooler; its temperature is reduced to a specified value. After passing through cooling coil, the pre-cooled air enters the dehumidifier and contacts the flowing cooled desiccant solution on the filer surface, initiating simultaneous heat and mass transfer process. During this interaction, both heat and moisture are transferred from air to desiccant solution. The air undergoes further cooling and dehumidification, ultimately reaching the specified temperature and humidity level. Meanwhile, the desiccant solution becomes diluted as it absorbs moisture. When its concentration drops below a predefined threshold, the desiccant solution will be transferred to the regeneration system for concentration, restoring its dehumidification capacity.

The cooling capacity, moisture removal rate, thermal efficiency and moisture effectiveness are commonly employed as performance indicators for evaluating the LDDS system in various studies [36,37,38] and are defined as follows:

(1) $$\begin{array}{cc} \; & Q_{cd}={\dot{m}}_{a}\left(H_{a,i}-H_{a,o}\right)\\ \; & N_{cd}={\dot{m}}_{a}\left(\omega_{a,i}-\omega_{a,o}\right)\\ \; & \eta_{h,cd}=\frac{T_{a,i}-T_{a,o}}{T_{a,i}-T_{s,i}}\\ \; & \eta_{m,cd}=\frac{\omega_{a,i}-\omega_{a,o}}{\omega_{a,i}-\omega_{cd,equ}} \end{array}$$

where ${\dot{m}}_{a}$ represents air mass flow rate; $T_{a,i}$, $T_{a,o}$, $H_{a,i}$, $H_{a,o}$, $\omega_{a,i}$ and $\omega_{a,o}$ denote air inlet and outlet temperature, enthalpy and humidity, respectively. $T_{s,i}$ is desiccant solution inlet temperature, and $\omega_{cd,equ}$ is desiccant solution equilibrium humidity ratio.

## 3. ITSO-TCN-BiGRU-SA Prediction Model

### 3.1. Temporal Convolutional Networks

The temporal convolutional network (TCN) is an innovative variant of CNN architecture that extends traditional one-dimensional CNN by incorporating three key components: dilated convolution, residual connections and causal convolution. In contrast to recurrent neural networks, TCN demonstrates superior capability in dealing with the issue of gradient vanishing and explosion.

#### 3.1.1. Causal Convolution

Causal convolution, a temporal modeling approach that enforces strict time constraints, can be mathematically expressed as follows:

(2) $$P\left(x_{t}\right)=\prod_{t=1}^{T}P(x_{t}|x_{1},x_{2},\ldots ,x_{t-1})$$

where *T* is total time, and P(·) is predicted probability.

**Figure 2:**
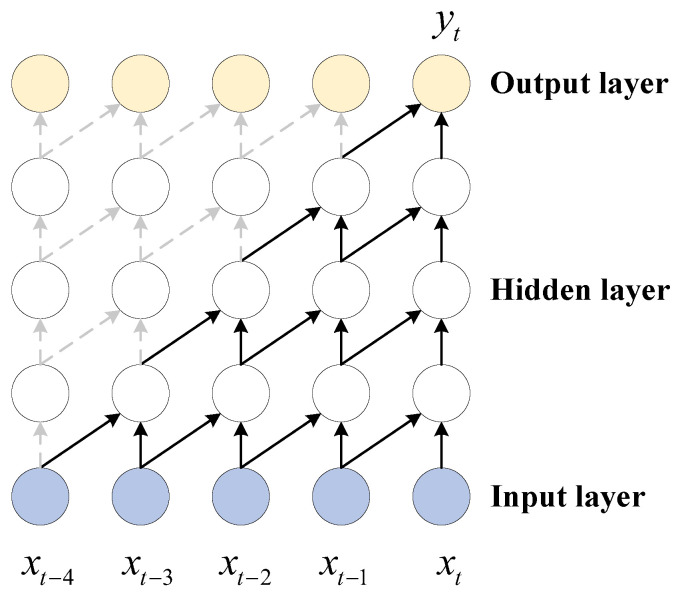
Schematic diagram of causal convolution.

The output sequence generated by causal convolution maintains identical length to its input sequence, while ensuring temporal causality by exclusively depending on current and preceding inputs, thereby effectively eliminating future information leakage. The schematic representation of this mechanism is illustrated in Figure 2.

#### 3.1.2. Dilated Convolution

**Figure 3:**
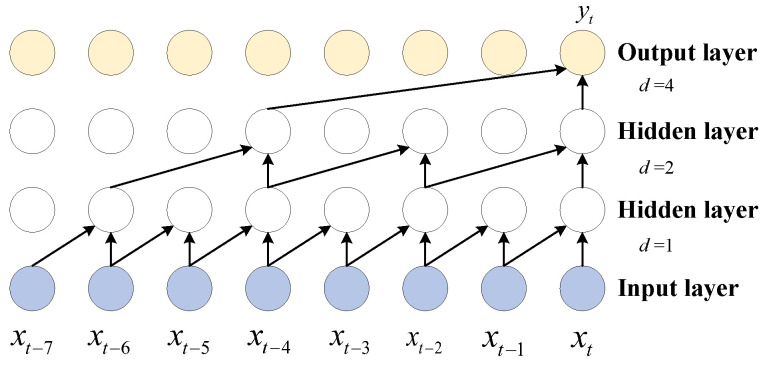
Schematic diagram of dilated convolution.

In contrast to standard convolution operations, dilated convolution significantly enhances the network’s receptive field efficiency by exponentially increasing the dilation rate across successive layers, thereby enabling TCN to achieve an extensive perceptual domain with minimal network depth. As illustrated in Figure 3, the expansion coefficient of exponential growth in the dilated convolution is $d_{i}=2^{i}$ (*i* is network layer number).

(3) $$F\left(s\right)=\left(x{*}_{d}f\right)\left(s\right)=\sum_{i=0}^{k-1}f(i)\cdot x_{s-d\cdot i}$$

where *k* is convolution kernel size, and *d* is expansion coefficient.

#### 3.1.3. Residual Module

**Figure 4:**
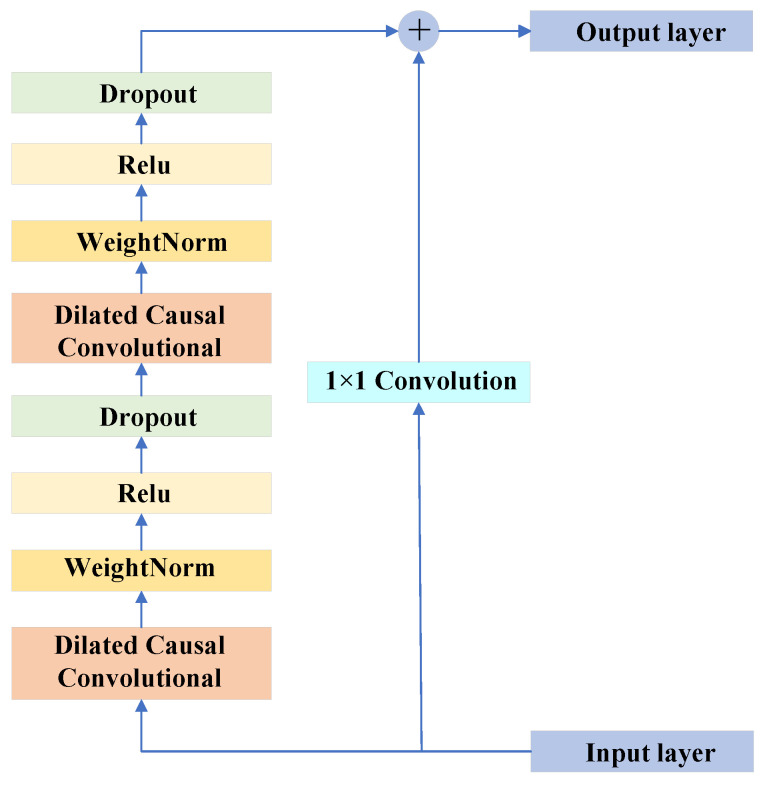
Schematic diagram of residual module.

A residual module is incorporated into the TCN model to mitigate information instability and loss issues caused by excessive network depth. The fundamental concept involves the integration of one or more layers with “skip connection” operation. The residual module comprises two dilated causal convolutional layers coupled with a nonlinear mapping and incorporates both weight normalization and Dropout techniques for network regularization. The architecture is illustrated in Figure 4. To address potential dimensionality mismatches between the input and output, an additional one-dimensional convolution is employed, ensuring that the tensors involved in the summation operation maintain consistent dimensions. The output of residual module is expressed as

(4) $$o=Activation\left(x+F\left(x\right)\right)$$

(5) $$F\left(s\right)=\left(x{*}_{d}f\right)\left(s\right)=\sum_{i=0}^{k-1}f(i)\cdot x_{s-d\cdot i}$$

### 3.2. Bidirectional Gated Recurrent Unit

The Gated Recurrent Unit (GRU) is a variant of RNN, derived as a simplification of the LSTM architecture. It was designed to address challenges such as vanishing gradients in long-term memory and backpropagation. Compared to LSTM, GRU offers a more compact architecture with significantly fewer parameters, while achieving comparable predictive accuracy. The mathematical formulation of GRU is as follows:

(6) $$\begin{array}{cc} \; & z_{t}=\sigma \left(W^{Z}x_{t}+U^{Z}h_{t-1}\right)\\ \; & r_{t}=\sigma \left(W^{r}x_{t}+U^{r}h_{t-1}\right)\\ \; & {\tilde{h}}_{t}=\tanh\left(r⊙Uh_{t-1}+Wx_{t}\right)\\ \; & h_{t}=\left(1-z_{t}\right)⊙{\tilde{h}}_{t}+z_{t}⊙{\tilde{h}}_{t-1} \end{array}$$

where $h_{t}$ and $r_{t}$ represent the state of hidden unit and reset gate, respectively; σ(·) stands for the sigmoid function and acts as an activation function. $W^{Z}$ and $U^{Z}$ are the weights of update gate, $W^{r}$ and $U^{r}$ are the weights of reset gate and of update gate.

**Figure 5:**
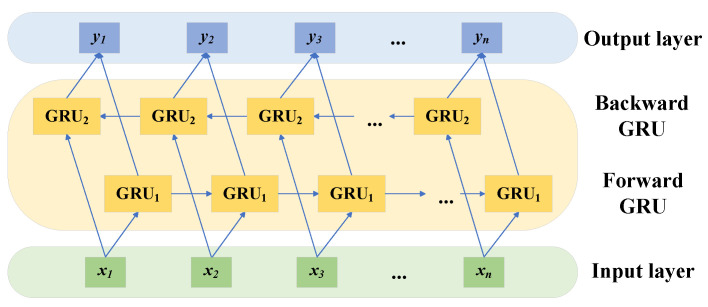
Schematic diagram of BiGRU.

Bidirectional Gated Recurrent Unit (BiGRU) is an advanced variant of GRU architecture. In this network, the output layer integrates information from both backward and forward propagation. Each training sequence is processed by two distinct hidden layers, one operating in a backward direction and the other in a forward direction, and their outputs subsequently combined at the same output layer. This bidirectional mechanism enables the output layer to capture comprehensive contextual information, incorporating both past and future states of each point in the input sequence. Compared to the unidirectional GRU model, BiGRU exhibits faster convergence, greater resistance to overfitting, and improved accuracy, as it leverages the dual weights of backward and forward states. The architecture of BiGRU is illustrated in Figure 5.

### 3.3. Self-Attention Mechanism Module

The self-attention mechanism (SA) [39] is an intuitive method of explanation that mimics the mechanisms of human vision. It is extensively utilized in a variety of deep learning applications, such as image analysis, natural language processing and load prediction. It has been found that the relevant time series information can be effectively preserved by adding attention mechanisms and weight allocation principles into the network model. The formula of SA is given as follows:

(7) $$Attention\left(Q,K,V\right)=Softmax\left(\frac{QK^{T}}{\sqrt{d_{k}}}\right)V$$

where *V*, *K* and *Q* are the value matrix, key matrix and query matrix, respectively. The effect of each query element on all key elements in the SA is computed by taking the dot product of *K* and *Q*. Then, the result is divided by $\sqrt{d_{k}}$. Finally, the attention weight of each position is obtained through the *Softmax* function, and the weighted sum is output.

### 3.4. Improved TSO Algorithm

#### 3.4.1. TSO Algorithm

The Tuna Swarm Optimization (TSO) algorithm proposed by Xie et al. [40] is a swarm intelligence optimization algorithm, inspired by the foraging behaviors of tuna schools, specifically parabolic foraging and spiral foraging. During the population initialization phase, the TSO algorithm generates an initial population randomly within the search space. Assuming that there are NP tunas in the tuna stocks, the mathematical formulation for initializing each tuna individual in algorithm is expressed as

(8) $$X_{i}^{\mathrm{int}}=rand\cdot \left(ub-lb\right)+lb,i=1,2,\cdots ,NP$$

where $X_{i}^{\mathrm{int}}$ is the initial tuna, with each tuna corresponding to a candidate solution in the TSO algorithm. *rand* represents a uniformly distributed random vector with values ranging from 0 to 1. lb and ub denote the lower and upper boundaries of the search space.

Two distinct foraging strategies are employed by tuna swarms: parabolic foraging and spiral foraging. The selection probability for each strategy is set at 50%. The spiral foraging strategy’s mathematical formulation is expressed as

(9) $$\begin{array}{cc} \; & \alpha_{1}=a+\left(1-a\right)\cdot \frac{t}{t_{\max}}\\ \; & \alpha_{2}=\left(1-a\right)-\left(1-a\right)\cdot \frac{t}{t_{\max}}\\ \; & \beta =e^{bl}\cdot \cos\left(2\pi b\right)\\ \; & l=e^{3\cos(\left(\left(t_{\max}+1/t\right)-1\right)\pi )} \end{array}$$

(10) $$X_{i}^{t+1}=\left\{\begin{array}{c} \begin{array}{cc} \begin{array}{c} \begin{array}{c} \alpha_{1}\cdot \left(X_{rand}^{t}+\beta \cdot \left|X_{rand}^{t}-X_{i}^{t}\right|\right)\\ +\alpha_{2}\cdot X_{i}^{t},i=1, \end{array}\\ \begin{array}{c} \alpha_{1}\cdot \left(X_{rand}^{t}+\beta \cdot \left|X_{rand}^{t}-X_{i}^{t}\right|\right)\\ +\alpha_{2}\cdot X_{i-1}^{t},i=2,3,\cdots ,NP, \end{array} \end{array} & ,if\;rand<\frac{t}{t_{\max}} \end{array}\\ \begin{array}{cc} \begin{array}{c} \begin{array}{c} \alpha_{1}\cdot \left(X_{best}^{t}+\beta \cdot \left|X_{best}^{t}-X_{i}^{t}\right|\right)\\ +\alpha_{2}\cdot X_{i}^{t},i=1, \end{array}\\ \begin{array}{c} \alpha_{1}\cdot \left(X_{best}^{t}+\beta \cdot \left|X_{best}^{t}-X_{i}^{t}\right|\right)\\ +\alpha_{2}\cdot X_{i-1}^{t},i=2,3,\cdots ,NP, \end{array} \end{array} & ,if\;rand\geq \frac{t}{t_{\max}} \end{array} \end{array}\right.$$

where, *a* is a constant that governs the degree to which tuna follow the optimal individual and previous individual during the initial stage; *b* is a uniformly distributed random number within range [0, 1]. $X_{best}^{t}$ denotes the current optimal individual, while $\alpha_{1}$ denotes a trend weight coefficient that regulates the tendency of tuna individuals to either swim toward the optimal individual or randomly select adjacent individuals. Additionally, $\alpha_{2}$ is a trend weight coefficient that controls the movement of tuna individuals toward the front. β serves as a distance parameter, determining the distance between tuna individuals and either the best individual or a randomly chosen reference individual.

The mathematical formulation of parabolic foraging strategy is expressed as

(11) $$X_{i}^{t+1}=\left\{\begin{array}{c} \begin{array}{cc} \begin{array}{c} X_{best}^{t}+rand\cdot \left(X_{best}^{t}-X_{i}^{t}\right)\\ +TF\cdot p^{2}\cdot \left(X_{best}^{t}-X_{i}^{t}\right), \end{array} & if\;rand<0.5 \end{array}\\ \begin{array}{cc} TF\cdot p^{2}\cdot X_{i}^{t}, & if\;rand\geq 0.5 \end{array} \end{array}\right.$$

(12) $$p={\left(1-\frac{t}{t_{\max}}\right)}^{\left(t/t_{\max}\right)}$$

where *TF* is a random variable that takes a value of either 1 or −1.

#### 3.4.2. ITSO Algorithm

In the initialization stage of tuna algorithm, the initial position of tunas is generated randomly, which often leads to the initial tunas clustering in similar or identical positions. This results in minimal diversity among individuals and insufficient coverage of the search space, significantly impairing the algorithm’s global search capability. To address this issue, this paper introduces an improved circle chaotic mapping to replace the random initialization of the population in the original algorithm. The proposed approach is defined by the following formula:

(13) $$N_{i+1}=\;\mathrm{mod}\;\left(3.8N_{i}+0.4-\left(0.7/3.8\pi \right)\sin\left(3.8\pi N_{i}\right),1\right)$$

where $N_{i}$ denotes chaotic particles.

The improved circle chaotic mapping operator is integrated into the TSO algorithm to ensure a more uniform distribution of initial tuna individuals within the search space. This enhancement significantly increases the population diversity of TSO, thereby facilitating the generation of more evenly distributed candidate solutions.

Weight parameters play a critical role in swarm intelligence optimization algorithms. Selecting appropriate weights can effectively balance the algorithm’s exploration and convergence capabilities. In the TSO algorithm, the weight parameters $\alpha_{1}$, $\alpha_{2}$ and *p* determine the degree to which a tuna individual follows the optimal individual. When the weight parameters are larger, the tunas exhibit a stronger tendency to follow the optimal individual, enabling the entire population to explore the search space more thoroughly. Conversely, when the weight parameters are smaller, the tunas follow the optimal individual to a lesser degree, causing them to swim within a confined area, which helps establish a localized search region around each individual. However, the optimization process of TSO is highly complex, and the linearly varying weight parameters $\alpha_{1}$, $\alpha_{2}$ and *p* fail to accurately reflect the actual dynamics of the algorithm’s optimization process. To address the disadvantages of linear weight variation, nonlinear adaptive weight strategies have been introduced to enhance swarm intelligence optimization algorithms [41]. Experimental results demonstrate that the nonlinear adaptive weight strategy outperforms the linear weight strategy in terms of optimization performance. Consequently, the weight coefficients $\alpha_{1}$, $\alpha_{2}$ and *p* are redefined as $\alpha_{1i}$, $\alpha_{2i}$ and $p_{i}$, respectively, and are expressed as follows:

(14) $$\begin{array}{cc} \; & \alpha_{1i}\left(t\right)=\alpha_{1ini}-\left(\alpha_{1ini}-\alpha_{1fin}\right)\cdot \sin\left(\frac{t}{\mu \cdot t_{max}}\cdot \pi \right)\\ \; & \alpha_{2i}\left(t\right)=\alpha_{2ini}-\left(\alpha_{2ini}-\alpha_{2fin}\right)\cdot \sin\left(\frac{t}{\mu \cdot t_{max}}\cdot \pi \right)\\ \; & p_{i}\left(t\right)=p_{ini}-\left(p_{ini}-p_{fin}\right)\cdot \sin\left(\frac{t}{\mu \cdot t_{\max}}\cdot \pi \right) \end{array}$$

where μ=2, $\alpha_{ini}$ and $\alpha_{fin}$ represent the initial and final value of α, respectively. The improved weight parameters $\alpha_{1i}$, $\alpha_{2i}$ and $p_{i}$ exhibit rapid changes in the early stages, enabling tuna individuals to closely follow the optimal individuals and thereby enhancing the global exploration capability of TSO. In the later stages, the weight parameters change at a slower rate, allowing tuna individuals to more actively explore their surrounding areas, which improves the local search ability of TSO.

For swarm intelligence optimization algorithms, avoiding local optima during the optimization process is a significant challenge. The Levy flight strategy is a powerful approach to enhance the global exploration capability of intelligent optimization algorithms. This strategy incorporates a movement mechanism that combines both short and long steps. By employing long steps at low frequencies, the algorithm can conduct extensive searches across the entire search space, while using short steps at high frequencies enables intensive local exploration within the immediate vicinity. To leverage these advantages, the Levy operator is integrated into the TSO algorithm to refine its population update strategy.

Levy’s flight mechanism is

(15) $$levy\left(s\right)=0.01\times \frac{u}{{\left|v\right|}^{1/\xi }},u∼N\left(0,\sigma^{2}\right),v∼N\left(0,1\right)$$

where ξ=1.5, and the variance σ is shown in Equation (16).

(16) $$\sigma ={\left(\frac{\Gamma \left(1+\xi \right)\sin\left(\frac{\pi \xi }{2}\right)}{\xi \cdot \Gamma \left(\frac{1+\xi }{2}\right)\cdot 2^{\frac{1-\xi }{2}}}\right)}^{1/\xi }$$

(17) $$\Gamma \left(x\right)=\int_{0}^{+\infty }e^{-t}t^{x-1}dt$$

The arithmetic expression of the enhanced spiral foraging strategy, incorporating nonlinear adaptive weighting mechanism and Levy operator, is presented as follows:

(18) $$X_{i}^{t+1}=\left\{\begin{array}{cc} \begin{array}{c} \alpha_{1i}\left(X_{rand}^{t}+Levy\left(s\right)\left|X_{rand}^{t}-X_{i}^{t}\right|+\alpha_{2i}X_{i}^{t}\right),i=1,\\ \alpha_{1i}\left(X_{rand}^{t}+Levy\left(s\right)\left|X_{rand}^{t}-X_{i}^{t}\right|+\alpha_{2i}X_{i-1}^{t}\right),i=2,\cdots ,NP \end{array} & if\;rand<\frac{t}{t_{\max}}\\ \begin{array}{c} \alpha_{1i}\left(X_{best}^{t}+\beta \left|X_{best}^{t}-X_{i}^{t}\right|+\alpha_{2i}X_{i}^{t}\right),i=1,\\ \alpha_{1i}\left(X_{best}^{t}+\beta \left|X_{best}^{t}-X_{i}^{t}\right|+\alpha_{2i}X_{i-1}^{t}\right),i=2,\cdots ,NP \end{array} & if\;rand\geq \frac{t}{t_{\max}} \end{array}\right.$$

### 3.5. ITSO-TCN-BiGRU-SA Model

**Figure 6:**
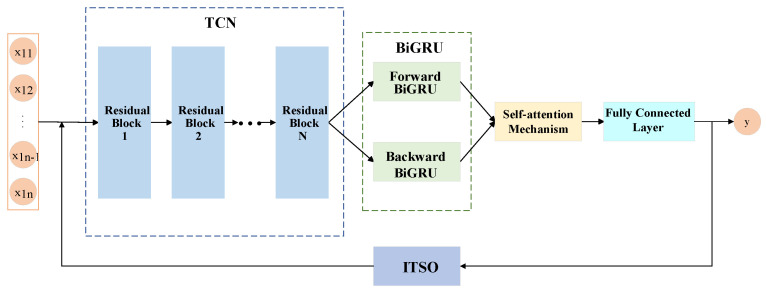
Schematic diagram of ITSO-TCN-BiGRU-SA.

The structure diagram of the proposed ITSO-TCN-BiGRU-SA prediction model is illustrated in Figure 6. The proposed ITSO-TCN-BiGRU-SA model architecture is a targeted design solution to address the core modeling challenges of the LDCD system. The LDCD system involves complex coupled heat and mass transfer of multiple fluids, such as air, solution, and cold water, in a two-stage refrigeration and dehumidification process. The temperature and humidity state of the outlet air is a result of the combined effects of multiple historical inlet parameters and current dynamics. This requires the predictive model to have the ability to capture local dynamics, understand long-term bidirectional dependencies, focus on key events, and handle high-dimensional parameter optimization. Therefore, the role of each component of the model in the prediction task is as follows: (1) TCN, through its causal dilated convolution structure, can efficiently and hierarchically extract local dependency patterns and short-term trends from the input parameter sequence (such as air temperature and humidity, solution flow rate and temperature, etc., over a period of time), which directly corresponds to the physical characteristic of the system state being affected by recent operating condition fluctuations. (2) BiGRU learns the long-term contextual dependencies of the sequence from both forward and backward dimensions, based on the local features extracted by TCN. This is crucial for simulating the LDCD system, as the current thermal and humidity state of the system is not only determined by past operating conditions but also contains an inertial trend towards a certain thermodynamic equilibrium. BiGRU can effectively model this global dynamic process. (3) SA is integrated to dynamically evaluate the importance of features at different historical moments. In LDCD operation, for example, the impact of step changes in solution concentration on current predictions is much greater than that during stable operation periods. The SA mechanism can adaptively assign higher weights to key time steps, allowing the model to “focus” on the transient processes that are most decisive for predictions, thereby improving the accuracy of characterizing the system’s nonlinear response. Finally, (4) the introduction of the ITSO algorithm aims to solve the inherent problem of the automatic optimization of high-dimensional hyperparameters in the aforementioned TCN-BiGRU-SA hybrid architecture. Manually tuning such a complex model is prone to falling into local optima and is inefficient. Through improvements in chaotic mapping initialization, nonlinear adaptive weights, and Levy flight strategies, the ITSO algorithm achieves intelligent and efficient global search in the model’s hyperparameter space, ensuring that this complex architecture can be automatically adjusted to its optimal performance state. It is a key guarantee for transforming the potential of theoretical models into practical high-precision predictions. In summary, each part of this model is tailored to the physical characteristics of LDCD system operation, collectively forming an adaptive collaborative prediction framework.

The TCN-BiGRU model is well-suited for processing long-sequence data, while the SA enhances the model’s ability to focus on critical information across varying time scales and mitigates issues such as gradient vanishing in long sequences [42]. By dynamically assigning self-attention weights to different time steps, the mechanism improves feature identification and extracts more meaningful representations from temporal data. Consequently, the TCN-BiGRU-SA model, which integrates the SA into the TCN-BiGRU framework, can selectively extract key features from various time steps, capture long-term dependencies and essential information within the sequence, and ultimately improve the model’s predictive performance.

If the setting parameters in the neural network are given manually, it is difficult to find the most suitable parameter values for the model. Therefore, the ITSO optimization algorithm is introduced into the TCN-BiGRU-SA model, and the learning rate, TCN expansion coefficient, filter number, BiGRU hidden layer neuron number, self-attention mechanism key value and regularization parameter, which have great influence on the performance of model, are optimized to find the most suitable parameter values. The mean absolute percentage error (MAPE) between the predicted and expected values is adopted as the fitness function, as defined by Equation (19).

(19) $$Y_{MAPE}=\frac{1}{n}\sum_{t=1}^{n}\frac{\left|y_{t}-{\hat{y}}_{t}\right|}{y_{t}}\times 100\%$$

The specific steps are as follows:

Step 1: Divide the dataset into training and test sets, and normalize the data.

Step 2: Optimize the hyperparameters. Define the lower and upper bounds of hyperparameters, set the initial tuna population size, and specify the maximum number of iterations. Generate the initial population position using Equation (13).

Step 3: Compute the fitness function of the ITSO algorithm and determine optimal tuna individual position based on the ITSO algorithm’s search principles.

Step 4: Set a random number rand; if *rand* < z, then use Equation (10) to initialize the position, otherwise use the improved foraging method to update the position. When *rand* < 0.5, the parabolic foraging strategy of Equation (11) is used to update the position, and the improved spiral foraging strategy of Equation (18) is used to update the position.

Step 5: Substitute the individual parameters of the updated position into the TCN-BiGRU-SA model, and save the predicted temperature and humidity results. Return to step 3 if the maximum number of iterations is not reached. Otherwise, output the optimal parameters and results and complete the algorithm iteration procedure.

## 4. Results and Discussion

### 4.1. Experimental Data and Network Parameters

**Figure 7:**
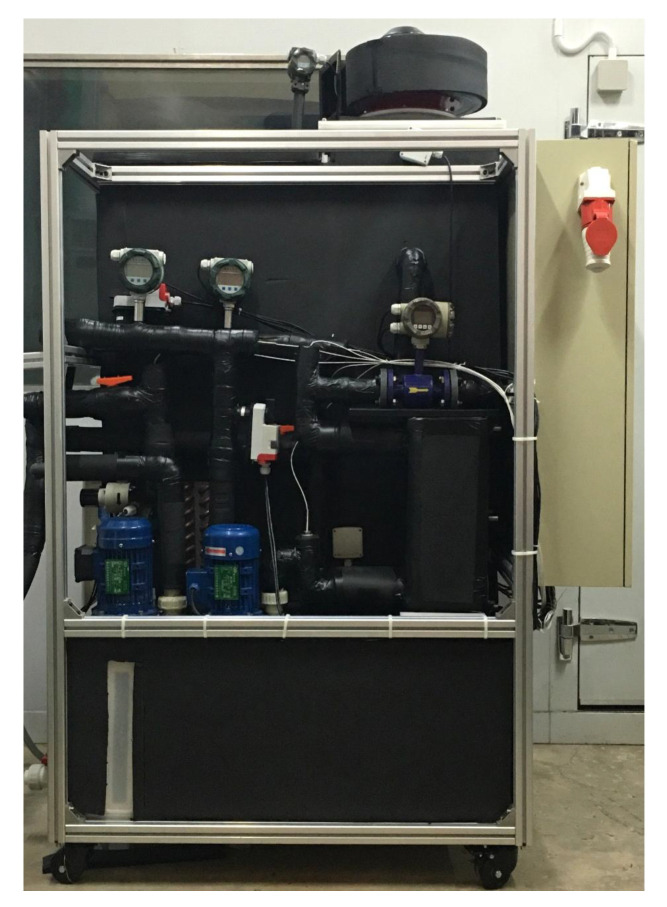
Pilot plant of LDCD system.

The experimental platform of the LDCD system is illustrated in Figure 7. The liquid desiccant used in the platform is lithium chloride solution. Key parameters, including the flow, temperature and humidity of air, flow and temperature of chilled water, and the flow, temperature and concentration of desiccant solution are measured and collected. Given that the outlet air temperature and humidity are influenced by multiple factors and considering the characteristics of the heat and mass transfer processes, the temperature and humidity predictions are conducted separately. The input features for air temperature prediction model include the inlet air temperature, humidity, and mass flow rate, as well as the mass flow rate and temperature of the chilled water and desiccant solution. For the air humidity prediction model, additional input features such as the desiccant solution concentration are included alongside the aforementioned parameters.

A total of 3268 sets of sample data were collected, with an interval of 10 s between each set of sample data. To avoid evaluation bias resulting from data leakage, we partitioned the dataset chronologically into training (80%) and testing (20%) sets, rather than using random partitioning. Although this approach may lead to slight differences in operating condition distributions between the training and testing sets, it better simulates real-world engineering scenarios where historical data is used to predict future operational states, thereby ensuring the practical utility and reliability of the evaluation results. Moreover, to eliminate dimensional differences and enhance computational efficiency, the sample data were normalized. The normalization formula is as follows:

(20) $$D_{n}=\frac{D-D_{\min}}{D_{\max}-D_{\min}}$$

where $D_{\min}$ and $D_{\max}$ are the minimum and maximum values in sample data, respectively.

The sliding window method is adopted to construct the input data sequence, with a window length of 8 time steps and a step size of 1, thereby achieving overlapping sampling between adjacent windows. The hardware operating environment for this experiment consists of an Intel(R) Core(TM) Ultra 5 125H processor (with a main frequency of 3.60 GHz) and 32 GB of RAM, and the operating system is Windows 11. The training and simulation of the proposed model are both completed on the Matlab R2023b software platform. Before network training, the basic network parameters of ITSO-TCN-BiGRU-SA are set. In this paper, the Adam optimizer is selected, ReLU function is selected as the activation function, a single-head self-attention mechanism is adopted, convolution kernel size is set to 5, Dropout coefficient is 0.1, and maximum training times is 100. The learning rate, expansion coefficient, number of filters, number of neurons in the BiGRU hidden layer, key value of the self-attention mechanism, and the regularization parameter are optimized by the ITSO algorithm. The initial tuna population size is set to 10, and the maximum iteration is set to 50. The optimal parameters identified by the ITSO algorithm for the temperature model and the humidity models are presented in Table 1 and Table 2, respectively.

### 4.2. Model Validation

**Figure 8:**
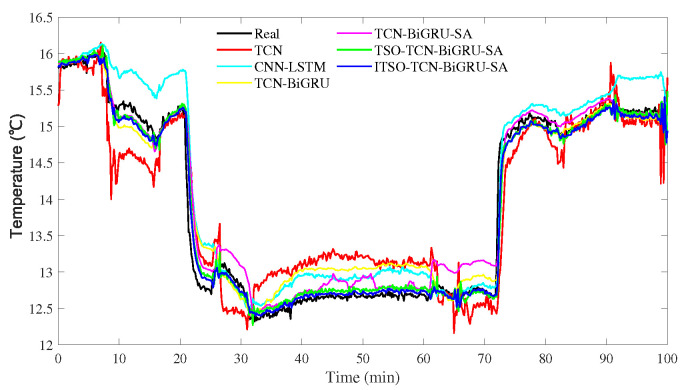
Comparison of air temperature model prediction curves.

**Figure 9:**
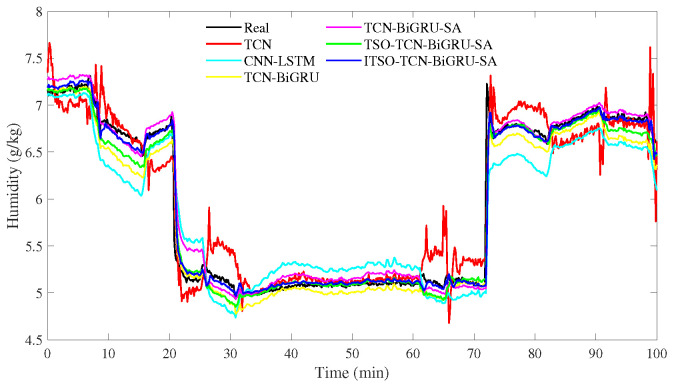
Comparison of air humidity model prediction curves.

**Figure 10:**
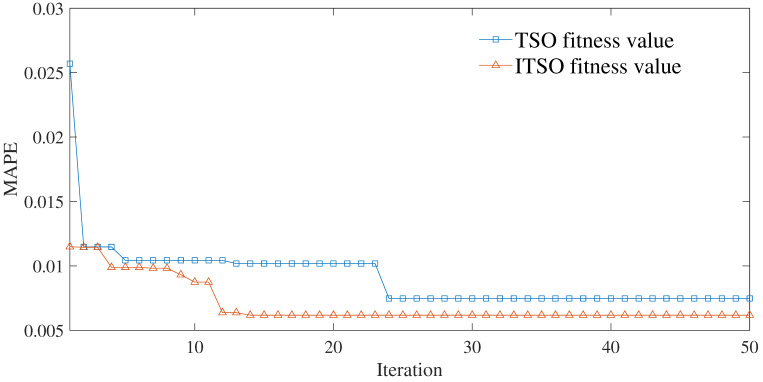
The fitness value curve of the air temperature model.

**Figure 11:**
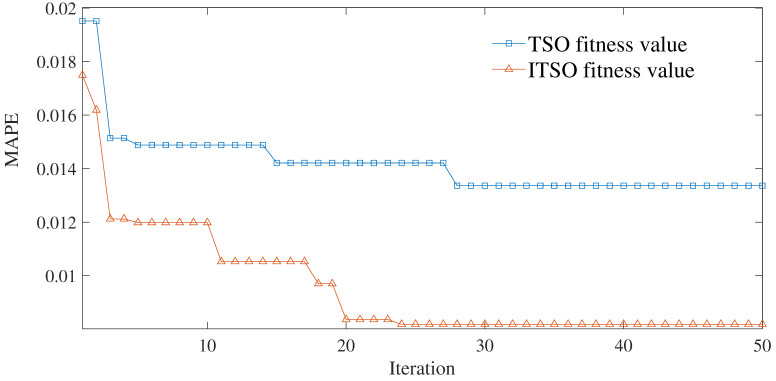
The fitness value curve of the air humidity model.

To validate the effectiveness of the proposed model, the TCN, CNN-LSTM, TCN-BiGRU, TCN-BiGRU-SA, TSO-TCN-BiGRU-SA models and the proposed ITSO-TCN-BiGRU-SA model were compared and verified in the temperature dataset and humidity dataset, respectively. The evaluation was performed using several widely recognized metrics, including mean absolute percentage error (MAPE), root mean square error (RMSE), mean square error (MSE), mean absolute error (MAE) and coefficient of determination ($R^{2}$). These metrics were employed to comprehensively assess the performance and accuracy of the developed models. The results are depicted in Figure 8, Figure 9, Figure 10 and Figure 11. The air temperature and humidity prediction curves of the different prediction models are illustrated in Figure 8 and Figure 9. The comparisons of fitness value curves of TSO-TCN-BiGRU-SA and ITSO-TCN-BiGRU-SA in the temperature model and humidity model are represented in Figure 10 and Figure 11. The error indexes are given in Table 3 and Table 4.

As illustrated in Figure 8 and Figure 9, the TCN-BiGRU model demonstrates superior prediction accuracy for both temperature and humidity compared to the standalone TCN model. In the TCN-BiGRU architecture, the TCN component effectively captures local features, while the BiGRU component enhances the model by leveraging bidirectional contextual relationships. This dual mechanism enables the model to gain a more comprehensive understanding of sequential data, thereby significantly improving prediction performance. Similarly, the prediction accuracy of the TCN-BiGRU-SA temperature and humidity models outperforms the TCN-BiGRU model. After adding the optimization algorithm, the MAPE of the TSO-TCN-BiGRU-SA temperature model decreased by 0.487% compared with TCN-BiGRU-SA, and $R^{2}$ increased by 1.606%. The MAPE of the ITSO-TCN-BiGRU-SA model decreased by 0.131% compared with TSO-TCN-BiGRU-SA model, and $R^{2}$ increased by 0.495%. The MAPE of the TSO-TCN-BiGRU-SA humidity model decreased by 0.27% compared with TCN-BiGRU-SA model, and $R^{2}$ increased by 0.651%. The MAPE of the ITSO-TCN-BiGRU-SA humidity model decreased by 0.518% compared with TSO-TCN-BiGRU-SA model, and $R^{2}$ increased by 0.698%. As depicted in Figure 10 and Figure 11, the ITSO algorithm exhibits a notably faster convergence speed compared to the TSO algorithm, while also achieving a smaller fitness value. This demonstrates the enhanced performance of the ITSO algorithm, which incorporates the nonlinear adaptive weights and Levy operator.

From Table 3 and Table 4, it is obvious that compared with the TCN, TCN-BiGRU, TCN-BiGRU-SA and TSO-TCN-BiGRU-SA temperature prediction models, the MAPE of the proposed ITSO-TCN-BiGRU-SA temperature model is reduced by 1.983%, 1.000%, 0.618%, and 0.131%, and $R^{2}$ is improved by 10.127%, 3.78%, 2.101%, and 0.495%, respectively; the MAPE of the proposed ITSO-TCN-BiGRU-SA humidity model is reduced by 1.972%, 1.240%, 0.788% and 0.518%, and $R^{2}$ improved by 4.778%, 2.219%, 1.349% and 0.698%, respectively. In summary, the proposed ITSO-TCN-BiGRU-SA model demonstrates superior prediction accuracy compared to the other models, thereby validating its effectiveness and robustness.

Although this study did not conduct large-scale multi-round repeated experiments, fixed random seeds were set during model training to ensure reproducible results. Observation of the training process revealed that the model converged smoothly within 100 epochs, and the loss curves of the training set and validation set were in good agreement, indicating stable model training without overfitting. For some sample points with relatively large prediction errors, verification showed that they mainly occurred during the transient stages of system startup or valve regulation, when the system was in a non-steady state, increasing the difficulty of instantaneous prediction. Once the system entered a steady state, the model’s prediction accuracy recovered rapidly.

### 4.3. Performance Investigation

**Figure 12:**
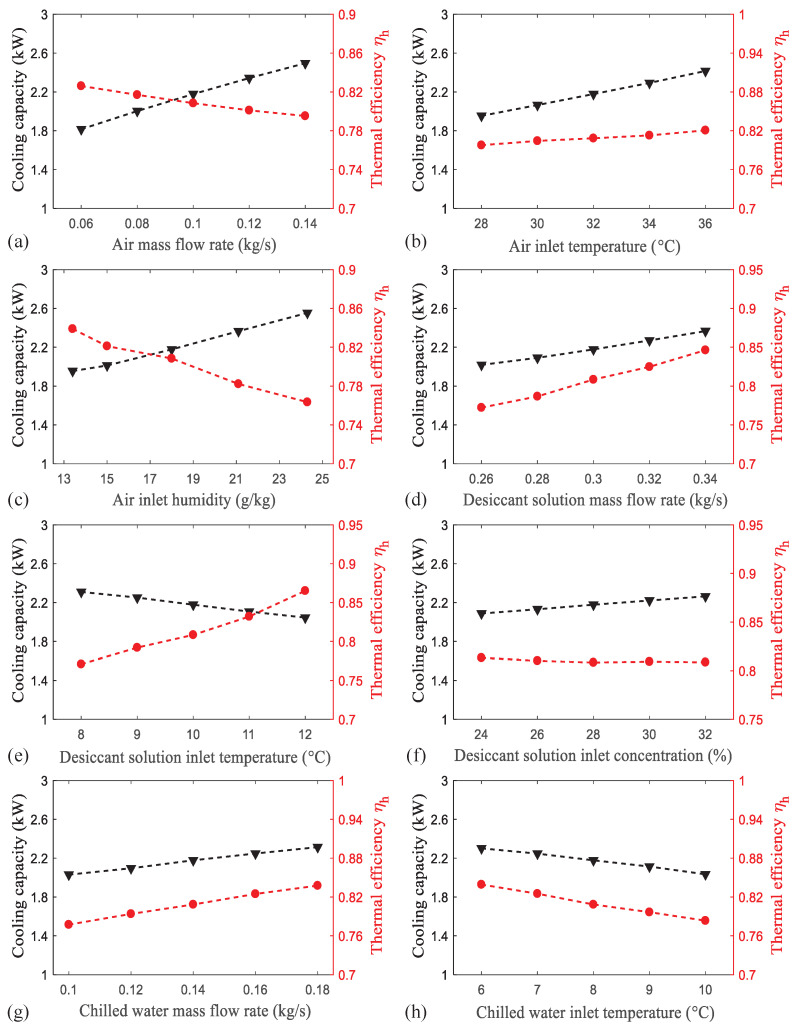
Influences of inlet parameters on cooling performance.

**Figure 13:**
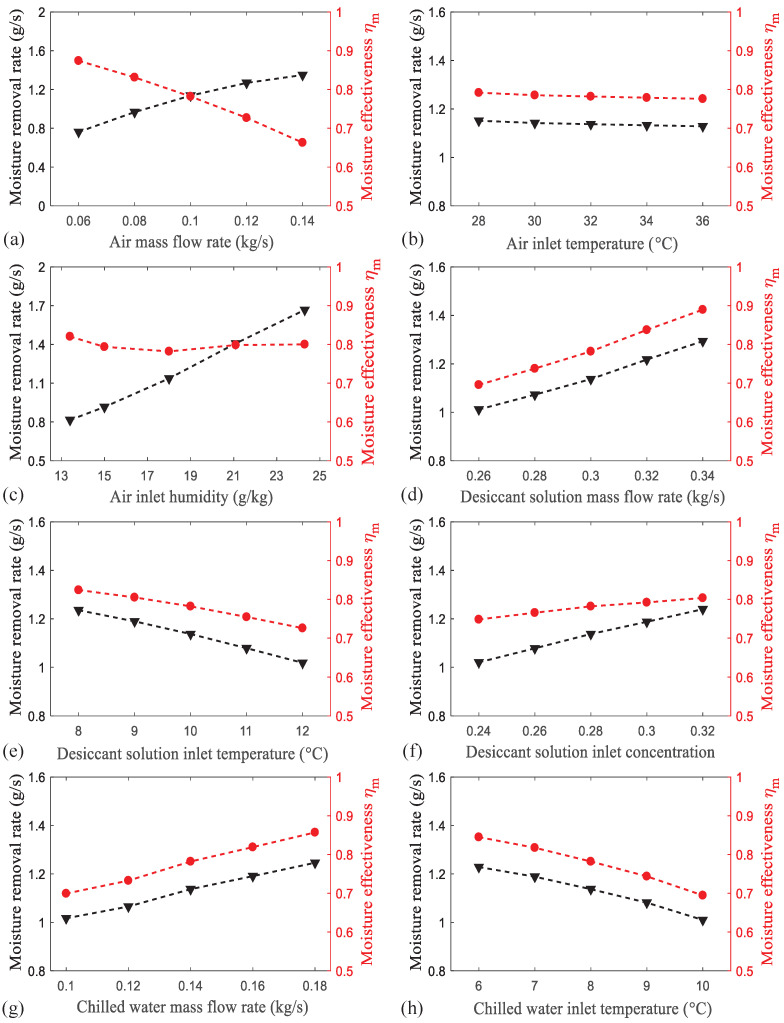
Influences of inlet parameters on the dehumidification performance.

In the LDDS system, the operating variables exert varying degrees of influence on the system’s cooling and dehumidification performance. To provide practical guidance for adjusting the system’s adjustable parameters, an analysis of how the relevant inlet parameters affect the system’s mass and heat transfer performance is conducted, based on the proposed ITSO-TCN-BiGRU-SA model. In each scenario, only one variable is modified while the others are maintained at the reference values. In the LDCD system, the cooling and dehumidification performance is affected by various inlet parameters, including the flow rate ${\dot{m}}_{a}$, ${\dot{m}}_{s}$, ${\dot{m}}_{w}$, inlet temperature $T_{a,i}$, $T_{s,i}$, $T_{w,i}$, humidity $\omega_{a,i}$ and concentration $\xi_{i}$. The operating ranges and reference values of the inlet parameters are provided in Table 5. The cooling and dehumidification performance are systematically compared and analyzed. The impact of operating parameters on the cooling capacity and thermal efficiency is illustrated in Figure 12, while their effects on dehumidification effectiveness and moisture removal rate are shown in Figure 13.

As illustrated in Figure 12 and Figure 13, the air inlet humidity, mass flow rate of air, desiccant solution and chilled water exhibit a positive effect on both the moisture removal rate and cooling capacity. In contrast, the desiccant solution and chilled water inlet temperature show a negative effect on both moisture removal rate and cooling capacity. The air inlet temperature positively influences cooling capacity but has minimal effect on the moisture removal rate. Similarly, the desiccant solution inlet concentration enhances the moisture removal rate but has negligible impact on the cooling capacity. Regarding thermal efficiency and moisture effectiveness, the mass flow rate of desiccant solution and chilled water demonstrates a positive influence, whereas the air mass flow rate and chilled water inlet temperature exhibit a negative effect. The air inlet temperature and desiccant solution inlet concentration have little to no effect on these metrics. The air inlet humidity negatively affects thermal efficiency but has minimal impact on moisture effectiveness. The desiccant solution inlet temperature positively influences thermal efficiency while negatively affecting moisture effectiveness.

## 5. Conclusions

This study aims to develop a data-driven model for the two-stage LDCD system to accurately predict the supply air temperature and humidity. The ITSO-TCN-BiGRU-SA hybrid prediction model constructed in this study integrates the TCN-BiGRU-SA network architecture with an improved TSO algorithm (incorporating the Levy operator and nonlinear adaptive weights). It demonstrates excellent performance in sequence modeling and prediction tasks, with high accuracy and strong applicability for predicting the supply air temperature and humidity of the LDCD system, thus supporting the optimization of control performance and energy efficiency improvement. Based on the proposed model, this study reveals that the influence of system inlet parameters on cooling and dehumidification performance is driven by the internal thermodynamics and heat-mass transfer mechanisms of the LDCD: the air inlet temperature affects the cooling capacity through the sensible-heat temperature difference, while the solution inlet temperature influences the dehumidification performance through the water vapor partial pressure; the air inlet humidity ratio affects both the cooling load and the mass-transfer process, and the solution concentration significantly enhances the moisture-absorption potential; and flow rate changes comprehensively act on the thermal–moistureexchange performance from the dimensions of load and efficiency. The research results provide practical guidance for the LDCD system: in design, prioritize ensuring a high-concentration solution to maintain strong dehumidification capacity; in operation, adopt a decoupled optimization strategy; and in the future, combine the parameter interaction model with advanced algorithms to achieve dynamic collaborative optimization of multi-loop parameters, minimizing the total energy consumption while meeting thermal and moisture demands.

## Figures and Tables

**Table 1:** Optimal parameters for air temperature prediction model.

Model Parameters	Range	Optimal Value
Learning rate	[0.0001, 0.01]	0.003
Number of filters	[1, 128]	3
Expansion coefficient	[1, 2, 4, 8, 16]	[1, 2, 4]
Number of neurons in BiGRU hidden layer	[1, 200]	79
Key value of SA	[2, 100]	4
Regularization parameter	[0.00001, 0.001]	0.00003

**Table 2:** Optimal parameters for air humidity prediction model.

Model Parameters	Range	Optimal Value
Learning rate	[0.0001, 0.01]	0.0036
Number of filters	[1, 128]	5
Expansion coefficient	[1, 2, 4, 8, 16]	[1, 2, 4]
Number of neurons in BiGRU hidden layer	[1, 200]	84
Key value of SA	[2, 100]	38
Regularization parameter	[0.00001, 0.001]	0.00018

**Table 3:** Model validated errors of the air temperature prediction models.

Models	RMSE	MAE	MAPE	$R^{2}$
TCN	0.4363	0.3519	2.60%	88.70%
CNN-LSTM	0.3539	0.2852	2.05%	92.56%
TCN-BiGRU	0.2888	0.2145	1.62%	95.05%
TCN-BiGRU-SA	0.2347	0.1648	1.24%	96.73%
TSO- TCN-BiGRU-SA	0.1676	0.103	0.75%	98.34%
ITSO- TCN-BiGRU-SA	0.1404	0.0865	0.62%	98.83%

**Table 4:** Model validated errors of the air humidity prediction models.

Models	RMSE	MAE	MAPE	$R^{2}$
TCN	0.2243	0.1664	2.79%	93.53%
CNN-LSTM	0.2912	0.2475	4.15%	89.08%
TCN-BiGRU	0.1744	0.1257	2.06%	96.09%
TCN-BiGRU-SA	0.1536	0.092	1.61%	96.96%
TSO- TCN-BiGRU-SA	0.1363	0.0805	1.34%	97.61%
ITSO- TCN-BiGRU-SA	0.1147	0.0486	0.82%	98.31%

**Table 5:** The operating parameters settings of the LDCD system.

Parameters		Reference	Range	Unit
Outdoor air	temperature	32	28–36	°C
	humidity ratio	18.0	13.4–24.3	g/kg
	flow rate	0.10	0.06–0.14	kg/s
Chilled water	temperature	8	6–10	°C
	flow rate	0.14	0.10–0.18	kg/s
Desiccant solution	temperature	10	8–12	°C
	flow rate	0.30	0.26–0.34	kg/s
	concentration	28	24–32	%

## Data Availability

The data presented in this study are available on request from the corresponding author.
